# Megadose Methylprednisolone (MDMP) for Hemangiomatosis

**DOI:** 10.4274/tjh.2012.55706

**Published:** 2012-12-05

**Authors:** Serhan Küpeli, Derya Çimen, Begül Yağcı Küpeli

**Affiliations:** 1 Diyarbakır Children’s Diseases Hospital, Department of Pediatric Oncology, Diyarbakır, Turkey; 2 Diyarbakır Children’s Diseases Hospital, Department of Pediatric Cardiology, Diyarbakır, Turkey; 3 Dicle University, School of Medicine, Department of Pediatric Oncology, Diyarbakır, Turkey

## REPLY

**In response to “Megadose Methylprednisolone (MDMP) for Hemangiomatosis” **

We are grateful to Dr. Özsoylu for his valuable comments on our case report concerning the treatment of hemangiomatosis with propranolol [1]. One of the issues raised by Dr. Özsoylu is regarding with the terminology used to describe hemangiomatous lesions. Although the term infantile hemangiomatosis could be used solely to describe the lesions in our patient, as Dr. Özsoylu pointed out, recent reports have frequently categorized hemangiomas as localized and segmental subtypes. Localized hemangiomas originate from a central focus, whereas segmental hemangiomas are clusters of hemangiomas with an extension corresponding to a recognizable or significant portion of a developmental segment, or involve a broad anatomic region of skin [2]. The segments in our patient with hemangiomas on the mandible, chin, bilateral preauricular region, neck, anterior region of the tongue, and left periorbital region approximate the mandibular and maxillary prominences described in medical embryology texts [3]. 

Systemic corticosteroids have been the mainstay of hemangioma treatment since the 1960s. Oral or intravenous MP and prednisone are effective for shrinking hemangiomatous lesions in infants. Cushingoid appearance and delayed healing of the cervical ulcerated lesion in our case were attributed to prolonged use of systemic MP. Recent studies have reported successful treatment of hemangiomas with the β-blocking agent propranolol, even in low birth weight infants [4,5]. Propranolol is now considered a first-line choice in many centers for the treatment of infantile hemangiomas and vasoconstriction; decreased expression of vascular endothelial growth factor and β-fibroblast growth factor genes, and triggering apoptosis in capillary endothelial cells are the proposed mechanisms of action. Our patient had been treated initially with intravenous MP 30 mg/kg/d at another hospital and after discharge the same dose was prescribed for daily divided administration. As a result, on admission to our hospital the patient had been taking MP 30 mg/kg/d for 2 months. Unfortunately, our patient was treated with MDMP without respecting ACTH-corticosteroid homeostasis, the significance of which was emphasized by Dr. Özsoylu [6]. We are in absolute agreement with Dr. Özsoylu in that when treating with MDMP the timing and duration of administration of MP are extremely important; the daily dose must be administered at 0600, either orally or intravenously over the course of 10-15 min.
